# Structurally Stable Hollow-Fiber-Based Porous Graphene Oxide Membranes with Improved Rejection Performance by Selective Patching of Framework Defects with Metal–Organic Framework Crystals

**DOI:** 10.1021/acsami.4c13400

**Published:** 2024-12-19

**Authors:** Farhad Moghadam, Chenxi Zhang, Zihao Li, Jianing Li, Mengjiao Zhai, Kang Li

**Affiliations:** Barrer Centre, Department of Chemical Engineering, Imperial College London, London SW7 2AZ, United Kingdom

**Keywords:** graphene oxide membranes, ZIF-8 crystals, targeted *in situ* growth, nanofiltration, framework defect patching

## Abstract

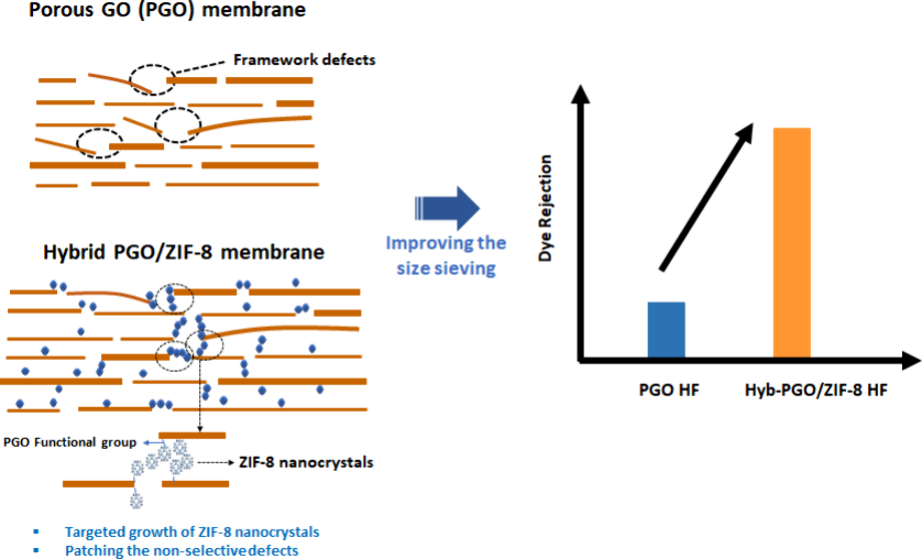

Graphene oxide (GO)-based membranes have demonstrated great potential in water treatment. However, microdefects in the framework of GO membranes induced by the imperfect stacking of GO nanosheets undermine their size-sieving ability and structural stability in aqueous systems. This study proposes a targeted growth approach by growing zeolitic imidazolate framework-8 (ZIF-8) nanocrystals precisely to patch microdefects as well as to cross-link the porous graphene oxide (PGO) flakes coated on the outer surface of the hollow fiber (HF) alumina substrate (named the hybrid PGO/ZIF-8 membrane). This method simultaneously improves their structural stability and size-sieving performance without compromising their water permeance. Various structural and elemental analyses were used to elucidate the targeted growth of the ZIF-8 crystals. The X-ray photoelectron spectroscopy (XPS) analysis confirmed the targeted coordination interaction of oxygen moieties on the edges of PGO nanosheets with metal ions of ZIF-8 crystals, allowing for the precise growth of the ZIF-8 nanocrystals in the PGO membranes. The XPS depth profile analysis revealed the uniform distribution of the ZIF-8 precursor throughout the PGO/ZIF-8 membrane. The resultant membrane showed a water permeance of 4 L m^–2^ h^–1^ bar^–1^ and maintained a very stable performance under pressure and prolonged cross-flow operation. Notably, the molecular weight cutoff (MWCO) improved considerably from 570 to 320 g/mol without sacrificing any water permeance after the targeted growth of ZIF-8. This research paves the way for the preparation of highly selective and stable PGO-based membranes for applications in industrial wastewater treatment.

## Introduction

Graphene oxide (GO)-based membranes with a laminar structure, precisely defined interlayer spacing, and tunable chemical functionality hold enormous potential for water separation applications.^1−3^ Inspired by ultrafast water transport through smooth graphitic capillaries, we initially expected that GO membranes would exhibit ultrahigh water permeance. However, the oxygen-based moieties on GO nanosheets create atomic-level roughness and friction on the walls of nanocapillaries, significantly hindering the ultrafast permeation rate.^4−6^ Theoretical studies show that GO nanosheets containing even a very low density of oxygen-based groups can strongly interact with water molecules. These interactions play a side-pinning role and significantly hinder the movement of water through confined capillaries.^7^ Moreover, high-aspect-ratio GO nanosheets assembled in the three-dimensional (3D)-layered structure form long and tortuous transport pathways. Notably, the tortuosity of transport pathways in GO membranes is about 3 orders of magnitude greater than that observed for typical porous media, significantly reducing the water permeance.^8^ The impact of tortuosity can be minimized by fabricating ultrathin GO membranes or using porous graphene oxide (PGO), i.e., creating additional pores on the surface of GO. In general, GO membranes exhibit water permeance ranging from 0.1 to 5 L m^–2^ h^–1^ bar^–1^ (LMHbar^–1^). Despite improvement in water permeance, GO-based membranes swell considerably in water due to their hydrophilic characteristics, which result from oxygen-containing moieties.^9,10^ This swelling behavior leads to a loose structure and poor size-sieving performance of the membranes. Cross-linking GO nanosheets with polymer compounds^9^ or ions^11^ is widely used to mitigate swelling. However, cross-linking the GO-based membranes results in a drop in permeance due to the reduction of the interlayer spacing. The imperfect stacking of GO nanosheets in the laminar structure is another issue that results in the formation of microdefects in the framework of GO membranes. These dynamic defects negatively impact the sieving ability and structural stability of GO membranes.^12,13^ Thus, developing new methods for fabricating GO-based membranes that offer (i) high stability and (ii) efficient rejection performance is critically important.

Different studies have been conducted to improve the rejection and structural integrity of GO membranes in the water.^2,14^ Banjerdteerakul et al.^15^ suggested using substrates with higher surface curvature to minimize the formation of wrinkles and microdefects in the layered structure of GO membranes. While this approach helped reduce the formation of microdefects, it did not fully address issues related to size exclusion and swelling. Zhang et al.^16^ employed an adsorption–nucleation–growth approach to grow zinc oxide (ZnO) nanoparticles at the interlayer of GO membranes, thereby generating reduced GO@ZnO composite membranes. The ZnO nanoparticles expanded the size of nanochannels and significantly enhanced the permeance of the composite membranes. However, the composite membranes showed 20 and 25% rejection rates for the small dyes methylene blue (MW = 319 g/mol) and methyl orange (MW = 327 g/mol), respectively, because ZnO nanoparticles created extra but non-selective permeation pathways in the layered structure of composite membranes. In this regard, metal–organic frameworks (MOFs) with uniform pore sizes and versatile chemistry have recently been employed to prepare stable composite membranes. Wang et al.^17^ utilized a mixed dispersion of copper-based MOF (Cu-TCPP) and GO nanosheets to prepare GO@Cu-TCPP composite membranes. The intercalated Cu-TCPP nanosheets enhanced the permeance of the resulting composite membranes, albeit with a low rejection rate, because of inefficient patching of GO framework defects. The same strategy was also employed to make GO@Cu-BDC and GO@zeolitic imidazolate framework-8 (ZIF-8) composite membranes.^18,19^ Zhang et al.^20^ prepared GO@ZIF-L composite membranes through a vacuum-assisted coating of a GO/zinc nitrate/1-methylimidazole dispersion. The GO@ZIF-L composite membranes were thick (6–8 μm) because ZIF-L nanosheets grew uncontrollably within the framework of the membrane. Simply mixing GO and MOF nanosheets to fabricate GO@MOF composite membranes undermines the unique size-exclusion characteristics of the MOF nanosheets. Therefore, achieving the selective and targeted growth of MOFs within the GO framework is crucial for realizing good structural stability and size-sieving ability. Recently, a two-step crystallization technique was proposed to grow ZIF-8 nanocrystals in the framework of GO, resulting in stable GO@ZIF-8 hybrid membranes.^21^ However, the molecular weight cutoff (MWCO) of charged dye molecules for the GO@ZIF-8 membrane was about 800, indicating that defects were not fully patched because of the non-selective growth of ZIF-8 nanocrystals. All of the aforementioned GO@MOF composite membranes demonstrated high water permeance because of the increased size of nanochannels. However, the size exclusion and structural stability of membranes remain a challenge. The proposed methods were not completely effective because MOFs could not be precisely intercalated or grown at targeted sites to patch non-selective microdefects, leading to poor structural stability and size exclusion of composite membranes.

The oxygen-containing groups, especially the hydroxyl groups located on the edges of GO flakes, serve as coordination sites for the directed growth of the MOFs. The negatively charged oxygen functional groups and positively charged metal ions can coordinately bond to selectively path the framework defect using MOFs. Here, we present a targeted growth method for growing ZIF-8 crystals on PGO flakes to fabricate hybrid PGO/ZIF-8 hollow fiber (HF) membranes (named Hyb-PGO/ZIF-8). The fabrication approach relies on utilizing the hydroxyl groups on PGO nanosheets to grow ZIF-8 nanocrystals on the edges of PGO nanosheets, achieving multiple objectives: patching the defects of PGO and stabilizing the structural integrity to prepare stable Hyb-PGO/ZIF-8 HF membranes with good dye rejection performance. Various characterization techniques, including X-ray diffraction (XRD), X-ray photoelectron spectroscopy (XPS), Fourier transform infrared spectroscopy (FTIR), energy-dispersive X-ray (EDX), and thermogravimetric analysis (TGA), were employed to confirm the targeted growth of the ZIF-8 crystals in the PGO framework. The resultant Hyb-PGO/ZIF-8 membrane, fabricated under optimized conditions, exhibited a water permeance of 4.0 LMHbar^–1^ and promising rejection of dye molecules in the range of 300–400 g/mol. Most importantly, the hybrid membrane demonstrated stable performance under pressure, highlighting the roles of ZIF-8 crystals in boosting the structural integrity of PGO membranes in a prolong operation.

## Experimental Section

### Materials

Alumina powder (99.99% purity, ultrapure grade) was provided by Inframat Advanced Materials LLC. Zinc nitrate hexahydrate, methanol, hydrochloric acid (HCl), ethanol, and hydrogen peroxide (H_2_O_2_) were obtained from VWR. Poly(methyl methacrylate) (PMMA) was supplied by Mitsubishi Chemical Group. Arlacel P135 was supplied by Croda. Potassium permanganate (KMnO_4_, ≥99.3%), natural graphite powder (325 mesh), and 2-methylimidazole (99%) were provided by Sigma-Aldrich. Ammonia solution (30%) and sulfuric acid (H_2_SO_4_, 98%) were supplied by Supelco. The glass fiber filter was purchased from Whatman. No further purification was done for the chemicals. Araldite epoxy adhesive was used to fix the hollow fiber substrate on the Swagelok tube.

### GO and PGO Synthesis

GO was produced according to our reported procedure.^22^ In brief, sulfuric acid (H_2_SO_4_) and then potassium permanganate (KMnO_4_) were gently added to graphite powder in an agitated two-wall glass reactor and thoroughly mixed at 35 °C overnight. After the GO suspension was diluted with water at 5 °C and hydrogen peroxide (H_2_O_2_), the dispersion was filtered and washed with diluted HCl. The resulting GO powder was dried in a desiccator for 3 days. Subsequently, dried GO was dispersed in acetone via bath sonication and filtered to remove any remaining acids, ensuring the high purity of GO. The GO powder was ready for homogeneous GO dispersion preparation after drying GO for at least 3 days under vacuum. The PGO dispersion was synthesized by the oxidative etching approach.^23^ A mixture of GO/NH_4_OH/H_2_O_2_ (20:1:1, vol) was prepared in a reactor with a controlled temperature (50 °C), and the reaction continued for 2–7 h. The PGO dispersion was centrifuged and purified using a dialysis membrane to remove any remaining NH_4_OH and H_2_O_2_. PGO nanosheets with etching times of 2, 3, 5, and 7 h, denoted PGO_2h, PGO_3h, PGO_5h, and PGO_7h, respectively, were synthesized in this study.

### Fabrication of Alumina Hollow Fibers

Hollow fiber supports were spun using a previously reported method.^24^

### Fabrication of PGO and Hy-PGO/ZIF-8 Hollow Fiber Membranes

**Figure 1:**
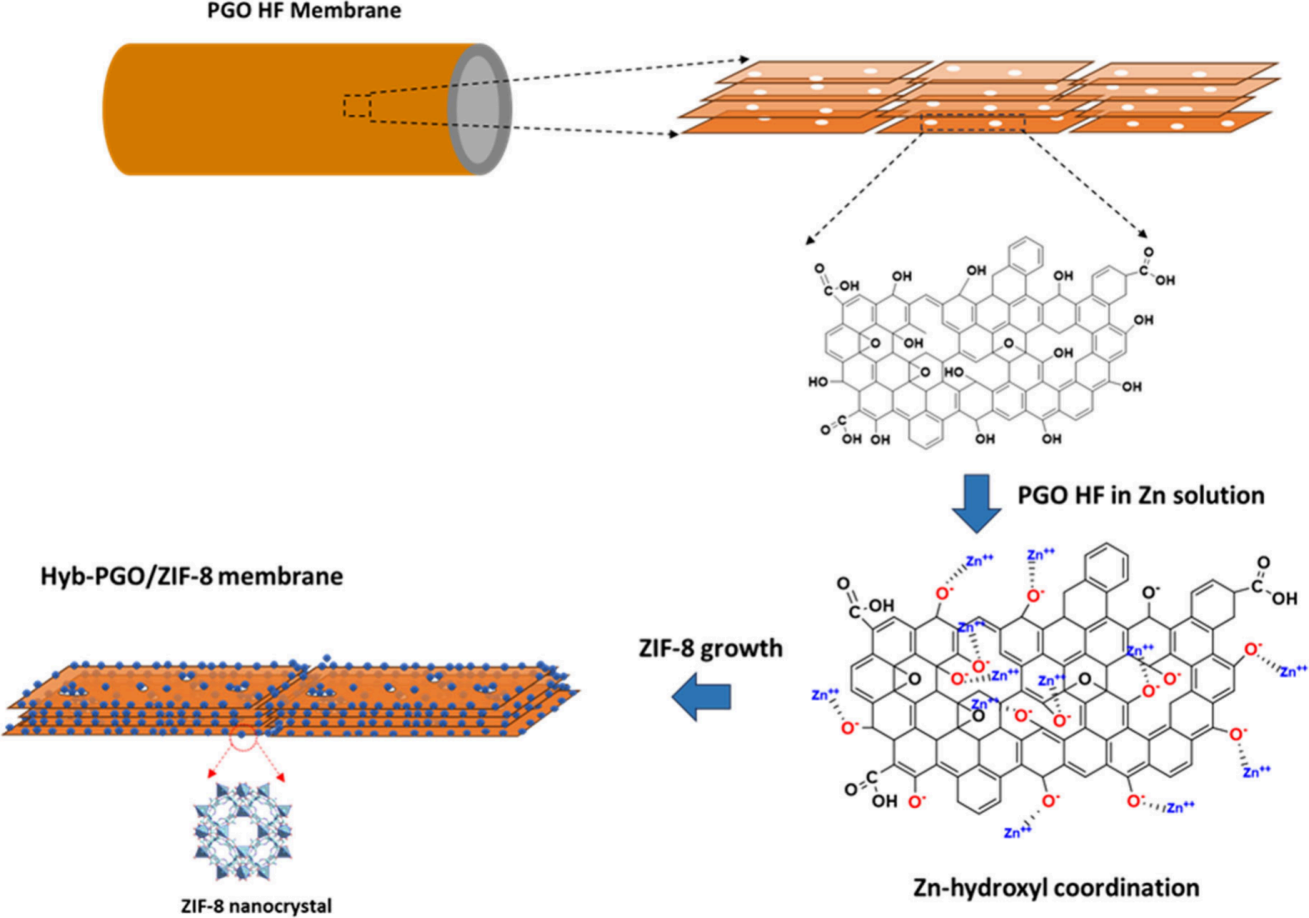
Schematic illustration of Hyb-PGO/ZIF-8 membrane fabrication via a two-step, targeted growth method.

The vacuum-assisted filtration technique was used to make PGO membranes.^15,22^ The thickness of the PGO layer was regulated by controlling the coating time. PGO hybrid membranes containing ZIF-8 crystals were prepared via a targeted growth technique, as shown in Figure 1. Initially, the PGO HF membrane was immersed in Zn(NO_3_)_2_·6H_2_O solution (3000 ppm) for 1–3 h (coordination step). Then, the metal-loaded PGO HF membrane was immersed in the ligand solution (i.e., 2-methylimidsazole) for a particular time to grow the ZIF-8 nanocrystals (*in situ* growth step). The resultant hybrid membranes were designated Hyb-PGO/ZIF-8 (*X*_*Y*), where *X* and *Y* represent the solvents used in the coordination and *in situ* growth steps, respectively. For example, the Hyb-PGO/ZIF-8 (W_M) membrane refers to the Hyb-PGO/ZIF-8 membrane prepared using a water-based solution of Zn(NO_3_)_2_·6H_2_O and a methanol-based solution of 2-methylimidsazole. Then, the Hyb-PGO/ZIF-8 membranes were put into pure methanol for 15 min and dried for 3 h. Various fabrication parameters, including the solvent used in both coordination and *in situ* steps, the duration of these steps, and membrane thickness, were optimized to fabricate Hyb-PGO/ZIF-8 with a high stability and good rejection performance without compromising the water permeance.

### Analytical Methods

The membrane morphology was studied using scanning electron microscopy (FEGSEM, LEO Gemini 1525). XPS was utilized to verify the reactions and determine the compositional changes during membrane fabrication. The X’Pert PAuminescent (voltage of 40 kV and current of 40 mA, 2θ = 5°–30°) was used to collect XRD spectra and determine the *d* spacing. The crystal loading in the hybrid membranes was measured by TGA. A Benchtop FTIR Cary spectrometer was used to collect the FTIR spectra.

### Dye Nanofiltration Performance

The water permeance and dye rejection was measured by a dead-end system. The PGO and Hyb-PGO/ZIF-8 HF membranes, fixed on a Swagelok tube, were placed into the cylinder filled with feed. The gas supplied to the upper part of the permeation cell regulated the system pressure. The mass of permeated water was recorded at 10 min intervals using a programmed balance, and the permeance *J* (L m^–2^ h^–1^ bar^–1^, LMHbar^–1^) was calculated as follows:
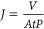
1In this equation, *P* refers to the operating pressure (bar), *t* is the time (h), *V* denotes the permeate volume (L), and *A* refers to the membrane area, which is from 1.4 × 10^–5^ to 2.2 × 10^–5^ m^2^. The operating pressure was adjusted to 7 bar, and the dye concentration was 20 mg/L. In this study, five different dyes, dispersal red (DR, 324.3 g mol^–1^), erythrosin B (EB, 835.9 g mol^–1^), methyl orange (MO, 327 g mol^–1^), crystal violet (CV, 408 g mol^–1^), and protoporphyrin IX (PPh-IX, 563 g mol^–1^), were utilized to assess the dye rejection. All tested samples were immersed in dye dispersion for 12 h to prevent dye adsorption from affecting the rejection performance. The permeate from the membrane was collected after 1 h to minimize the impact of extra adsorption of the membrane under pressure.

The dye rejection (*R*) was evaluated as follows:
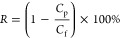
2The feed concentration (*C*_f_) and permeate concentration (*C*_p_) were determined with an ultraviolet–visible (UV–vis) spectrophotometer.

## Results and Discussion

### Structural Characterization

**Figure 2:**
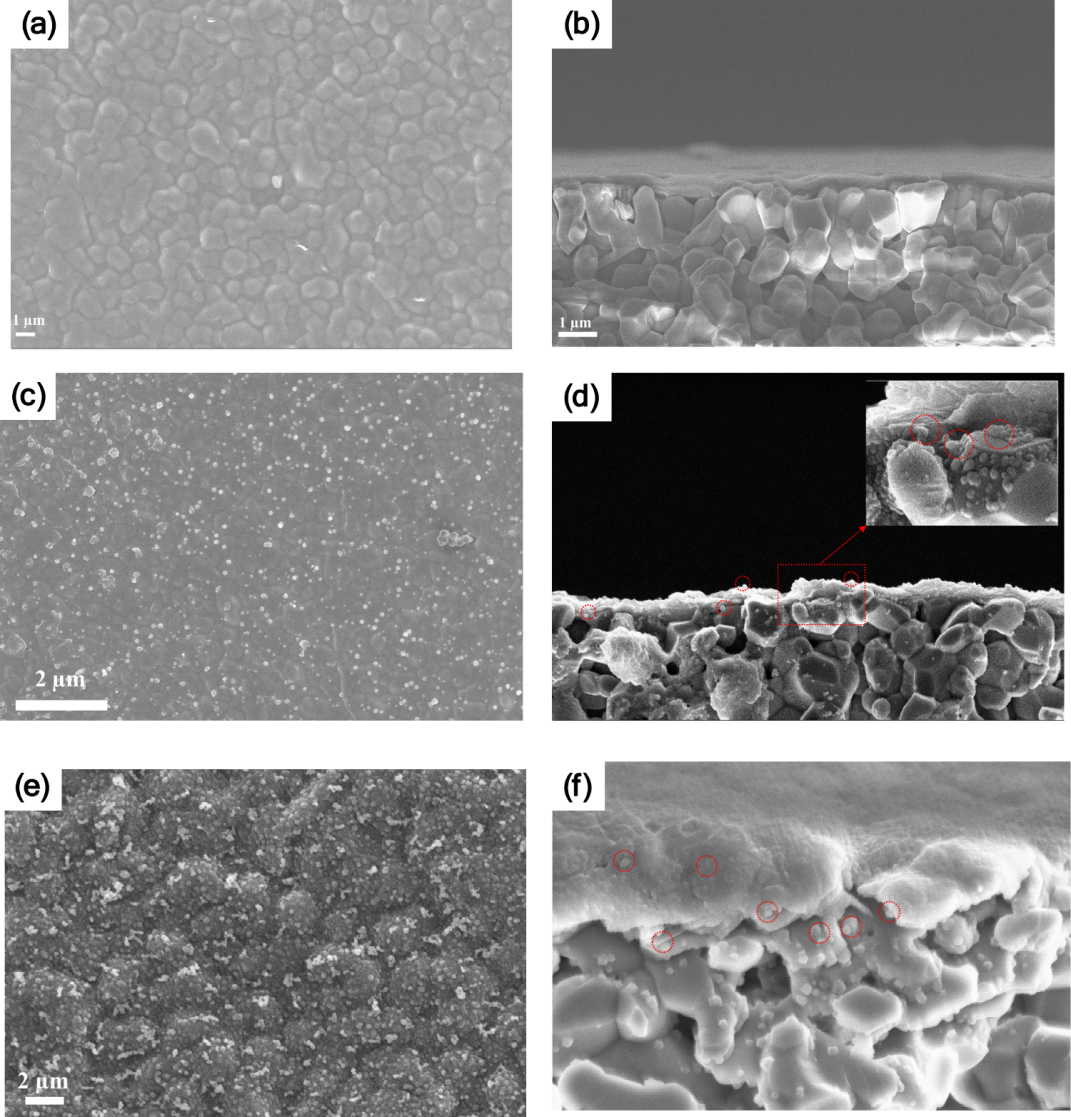
(a, c, and e) Surface and (b, d, and f) cross-sectional SEM images of the PGO and Hyb-PGO/ZIF-8 membranes. (a and b) PGO HF membrane. (c–f) Hyb-PGO/ZIF-8 HF membranes prepared using different solvents. (c and d) Methanol was used as the solvent in both steps, and (e and f) water and methanol were used as solvents in the coordination and *in situ* growth of ZIF-8, respectively. The red circles show the ZIF-8 nanocrystal grown in the layered structure of membranes. The PGO coating time was 30 s. The metal and ligand concentrations in the solvents were 3000 and 32 000 ppm, respectively.

Figure 2 displays the morphologies of the pristine PGO and Hyb-PGO/ZIF-8 HF membranes. The PGO HF membrane showed a smooth surface and a uniform laminar structure (panels a and b of Figure 2). The Hyb-PGO/ZIF-8 (M_M) HF membrane exhibited a uniform structure containing ZIF-8 nanocrystals with a controlled density within the PGO HF membrane framework (panels c and d of Figure 2). The average size of ZIF-8 nanocrystals on the surface ranges from 30 to 100 nm (Figure S1a of the Supporting Information). However, it should be noted that the size of the ZIF-8 nanocrystal within the framework of the membrane is smaller due to the confined space. In contrast, when water was used in the coordination step, ZIF-8 crystals with higher density were formed on the surface and within the framework of the Hyb-PGO/ZIF-8 (W_M) hybrid membrane, as shown in panels e and f of Figure 2 (Figure S1b of the Supporting Information). In particular, the surface density of ZIF-8 crystals on the membranes is notably high, primarily because the nucleation and crystallization rates were not effectively controlled. This behavior can be attributed to the swelling of PGO membranes in water, which facilitates the penetration of more metal ions, resulting in a higher density of ZIF-8 nanocrystals within the membrane framework. The impact of water on the ZIF-8 crystal growth was more pronounced when used in both steps, leading to the formation of larger crystals with different morphologies on the surface of the Hyb-PGO/ZIF-8 (W_W) hybrid membrane (Figure S2 of the Supporting Information). The growth pattern and morphology of ZIF-8 crystals depend upon solvent properties, such as polarity, viscosity, and interfacial tension between the solvent and solutes (metal and ligand).^25−27^ Methanol was found to be a more suitable solvent to moderate the nucleation and growth rates of ZIF-8 nanocrystals.^27^ This is aligned with our results that show that the growth of ZIF-8 nanocrystals can be controlled using methanol. The controlled growth of ZIF-8 crystals not only minimizes the resistance to water permeation through Hyb-PGO/ZIF-8 membranes but also facilitates the targeted patching of framework defects and improves the size-sieving performance. These aspects will be thoroughly discussed later.

The morphology of ZIF-8 nanocrystals in Hyb-PGO/ZIF-8 (M_M) differs significantly from that in Hyb-PGO/ZIF-8 (W_M), particularly in cross-sectional images. This variation is presumably due to the higher loading of Zn metal ions in Hyb-PGO/ZIF-8 (W_M), which results from higher swelling during the coordination step. Consequently, the lower ligand/metal ratio during the *in situ* growth step significantly influences the morphology of the ZIF-8 crystals. These results are consistent with a previously reported study.^28^

**Figure 3:**
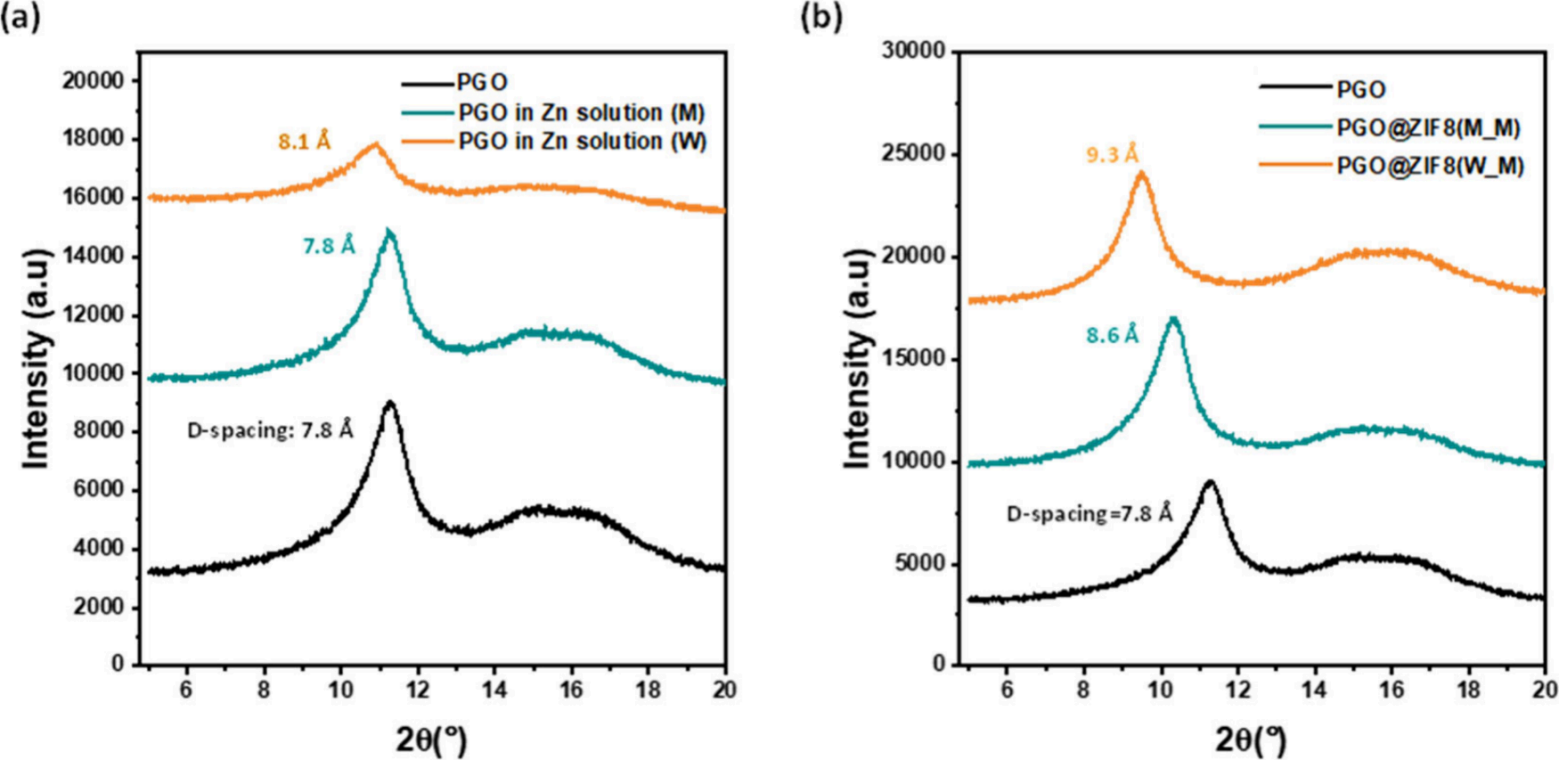
XRD patterns of (a) pristine PGO and PGO films immersed in water (W)- and methanol (M)-based zinc solutions and (b) resulting Hyb-PGO/ZIF-8 membranes. The concentrations of the zinc and ligand solutions were 3000 and 32 000 ppm, respectively.

The solvent significantly impacts both the coordination and growth steps in the fabrication of the Hyb-PGO/ZIF-8 membrane. During the coordination step, the loading of impregnated metal ions into the framework of the PGO HF membrane is controlled. The *d* spacing of the PGO HF membrane increased from 7.8 to 8.1 Å after immersion in a water solution of metal due to swelling. This swelling of the PGO HF membrane induces the non-controllable impregnation of metal ions within the framework of the membrane. In contrast, the interlayer spacing remained constant for the membrane immersed in the methanol solution (Figure 3a). During the *in situ* growth step, the solvent controlled the formation of crystals. Methanol, in particular, balances the nucleation and growth rates, resulting in smaller and more uniformly sized crystals. The *d* spacing of the Hyb-PGO/ZIF-8 (M_M) membrane is smaller than that of the Hyb-PGO/ZIF-8 (W_M) membrane (8.6 Å versus 9.3 Å), as shown in Figure 3b. This suggests that methanol effectively controls the crystal growth behavior within the framework. These results align well with previous studies highlighting how the solvent affects the crystal formation and morphology of ZIF-8.^27,29,30^ It is worth noting that the ZIF-8 corresponding peaks were not detectable in XRD spectra. These peaks may be relatively small due to the low loading of ZIF-8 in the hybrid membrane, causing them to overlap with the broad peak associated with PGO. Additionally, the thin membrane and high surface curvature of the substrate make it difficult to detect ZIF-8 peaks.

**Figure 4:**
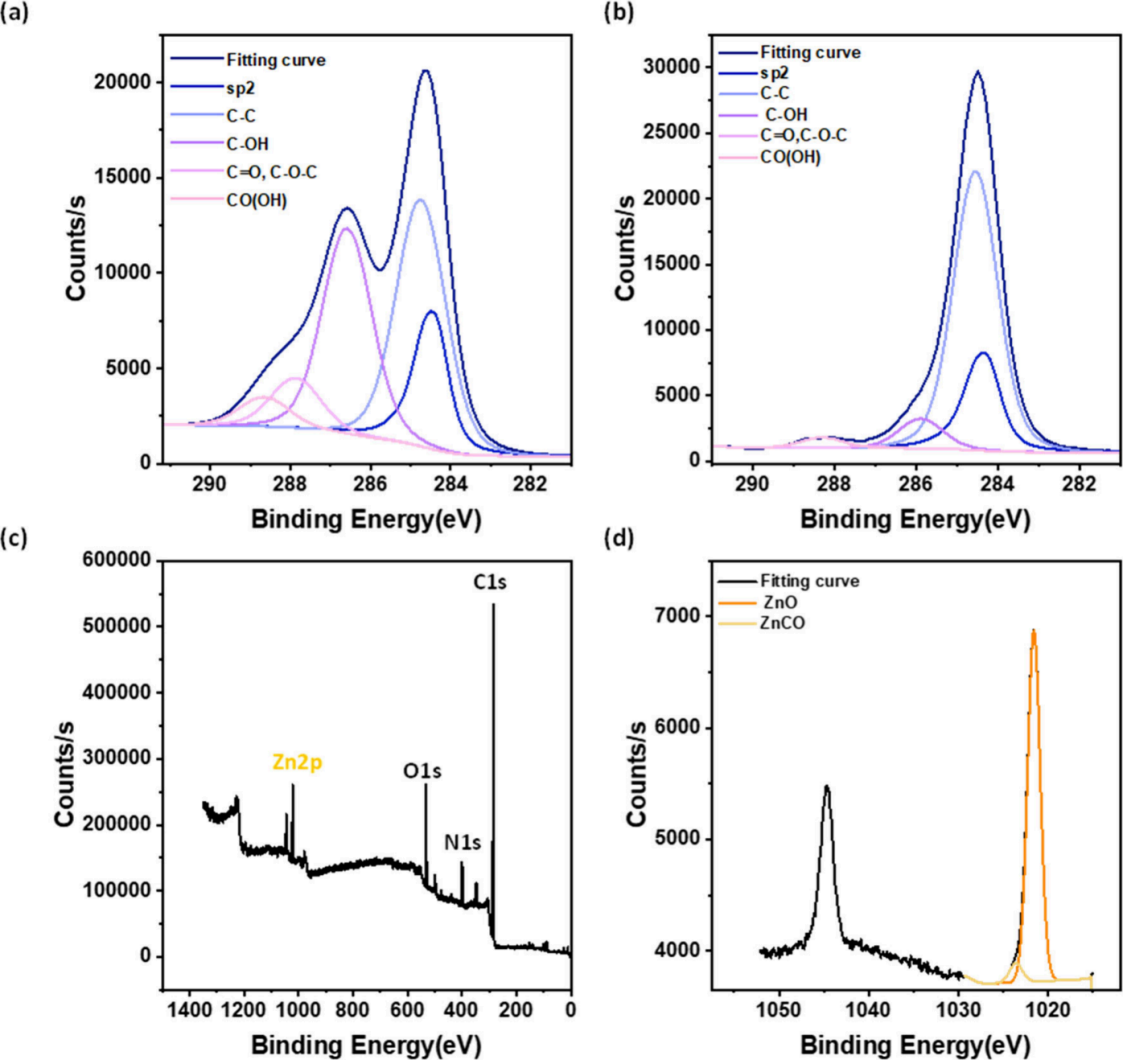
XPS C 1 s spectra of (a) pristine PGO and (b) Hyb-PGO/ZIF-8. (c) XPS survey and (d) Zn 2p spectra of the Hyb-PGO/ZIF-8 membrane. The metal and ligand concentrations were 3000 and 32 000 ppm, respectively.

The preferential growth of crystals within the PGO framework was realized through coordination interactions between the metal salt (zinc) and oxygen moieties. The C 1s spectra of the pristine PGO and Hyb-PGO/ZIF-8 (M_M) hybrid membranes revealed a noticeable decrease in the intensity of the C–OH (hydroxyl group) peak for the hybrid membrane. The bonding energy shifted from 288.6 to 288.2 eV in the hybrid membranes (panels a and b of Figure 4), indicating the interaction of hydroxyl groups on PGO nanosheets with zinc metal ions. This coordination band was further confirmed by the Zn 2p survey and spectra of the Hyb-PGO/ZIF-8 membranes (panels c and d of Figure 4), where two peaks were observed at 1021.6 and 1023.5 eV, corresponding to Zn–O and Zn-CO (Zn metal and hydroxyl groups) bonds, respectively. Furthermore, unlike that of the pristine PGO membrane, a C=N peak was detected in the N 1 s spectra of the Hyb-PGO/ZIF-8 membrane, which was assigned to the imidazole linkers of the ZIF-8 nanocrystals, thus confirming ZIF-8 nanocrystal growth in the hybrid membrane (Figure S3 of the Supporting Information). The uniform distribution of ZIF-8 nanocrystals within the layered structure of Hyb-PGO/ZIF-8 membranes was further proven by XPS depth profile analysis (Figure S4 of the Supporting Information). The relatively constant intensity of Zn 2p and N 1s peaks in the XPS spectra implies that the ZIF-8 precursor was fully and uniformly distributed and penetrated throughout the membrane framework.

**Figure 5:**
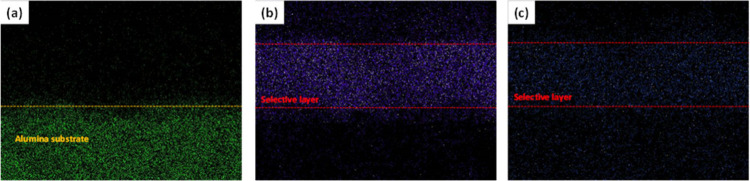
EDX mapping images of the Hyb-PGO/ZIF-8 membrane: (a) Al, (b) O, and (c) Zn.

**Figure 6:**
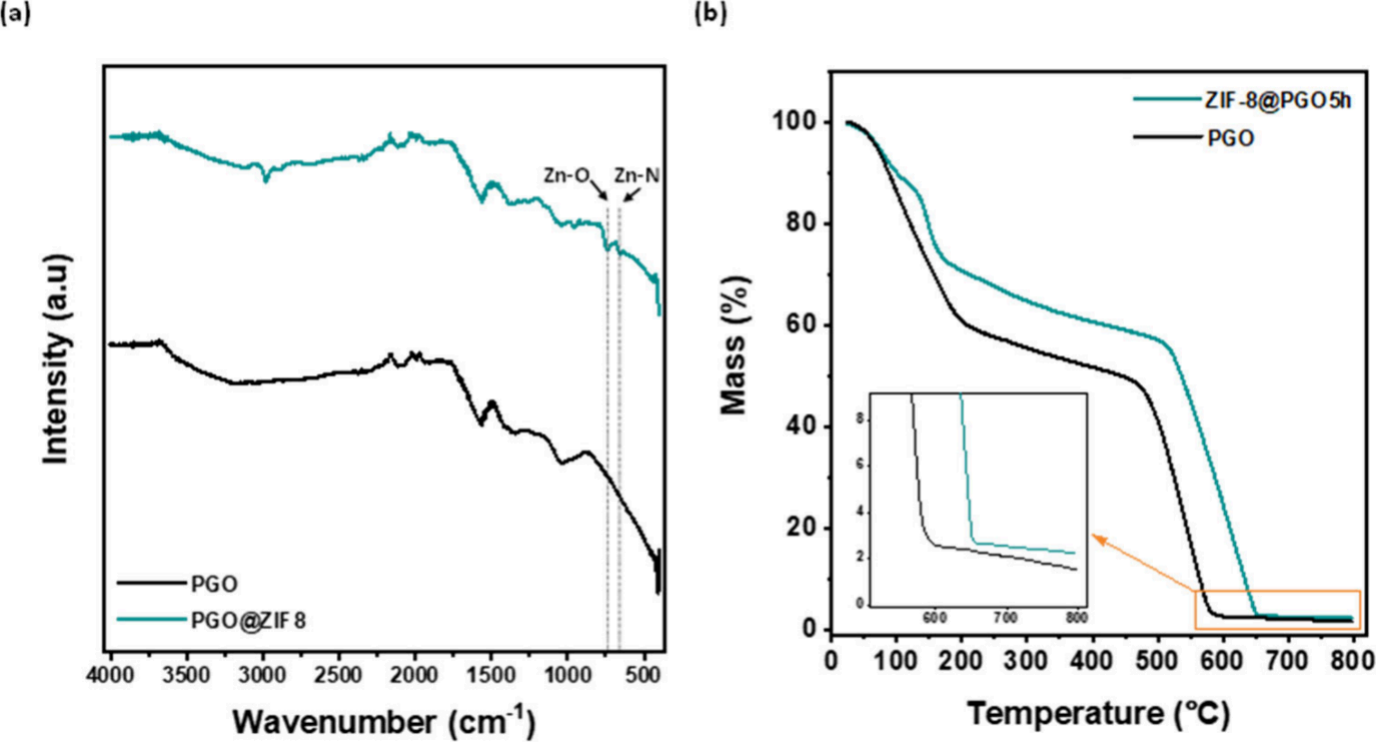
(a) FTIR spectra and (b) TGA results of the PGO and Hyb-PGO/ZIF-8 films. The Hyb-PGO/ZIF-8 film was prepared by immersion in a zinc solution (3000 ppm) and a ligand solution (32 000 ppm) for 3 h.

The EDX mapping images of Zn, O, and Al further confirmed that ZIF-8 crystals grew on the surface and in the layered structure of the Hyb-PGO/ZIF-8 membrane (panels a–c of Figure 5). Oxygen present in the cross-section of the hybrid membrane corresponds to oxygen moieties on the edges of the PGO nanosheets and the adsorbed metal salt (Figure 5b). A lower loading of zinc was detected within the PGO framework, but the loading was relatively low, as expected (Figure 5c). The FTIR spectra revealed details about the chemical composition of both the pristine PGO and Hyb-PGO/ZIF-8 membranes (Figure 6a). The characteristic bands at 741 and 666 cm^–1^ correspond to Zn–O and Zn–N vibrations, respectively, verifying the interaction of the zinc metal with the PGO functional group and ligand. The loading of 1.94 wt % of the ZIF-8 nanocrystals in the Hyb-PGO/ZIF-8 membrane was measured using TGA (Figure 6b).

According to the proposed models, the hydroxyl moieties are primarily found on the edges of GO nanosheets.^31^ Structural analysis confirmed that Zn metals coordinate with hydroxyl groups, serving as targeted positions for the growth of the ZIF-8 nanocrystals. Therefore, most of the crystals form at the edges of the PGO nanosheets. It is also anticipated that ZIF-8 nanocrystals will grow on the edges of pores containing hydroxyl groups. These ZIF-8 nanocrystals can play a dual role: they can patch the non-selective framework and create additional interactions between PGO nanosheets, thereby enhancing the stability of Hyb-PGO/ZIF-8 membranes. Moreover, the uniform pore size of ZIF-8 nanocrystals can enhance the rejection of hybrid membranes, a topic that will be further discussed in the subsequent section.

### Dye Nanofiltration Performance of the Membranes

**Figure 7:**
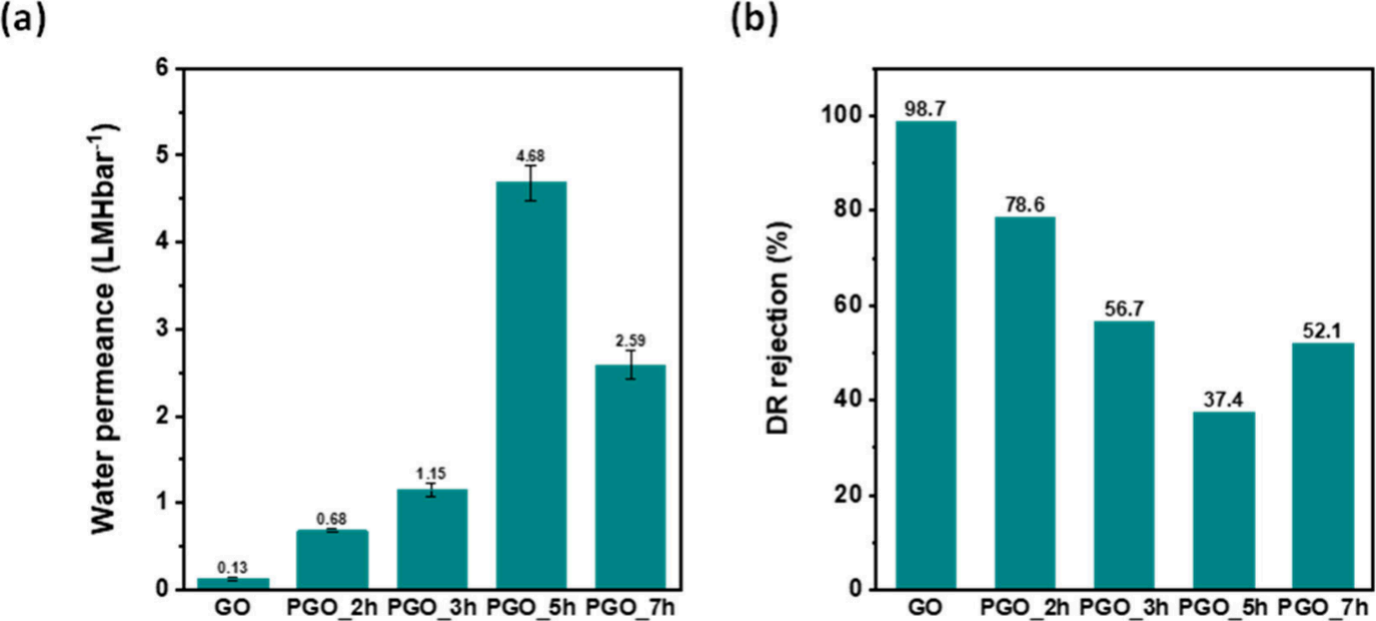
(a) Water permeance and (b) DR rejection of PGO samples as a function of etching times. A 0.1 mg/mL PGO dispersion and 30 s coating time were used for membrane preparation.

A pristine PGO HF membrane with a relatively high permeance is required to fabricate Hyb-PGO/ZIF-8 membranes with good structural stability and rejection performance. This facilitates the impregnation of metal ions and ligands into the framework of PGO HF membranes to grow ZIF-8 nanocrystals. Therefore, PGO nanosheets synthesized at different etching times were used to prepare pristine PGO HF membranes. A dead-end filtration system was used to assess the performance of samples.^22^ The water permeance was considerably improved by increasing the etching time to 5 h (Figure 7a). A longer etching time results in the creation of larger pores, providing shorter and less tortuous transport channels within the framework of membranes, consequently leading to greater water permeance.^22^ However, the permeance of the PGO_7h sample decreased by 45% compared to the PGO_5h sample because of the smaller *d* spacing, as evidenced by the higher DR rejection (Figure 7b). Therefore, the PGO_5h sample was chosen for the fabrication of Hyb-PGO/ZIF-8 membranes due to its high water permeance.

**Figure 8:**
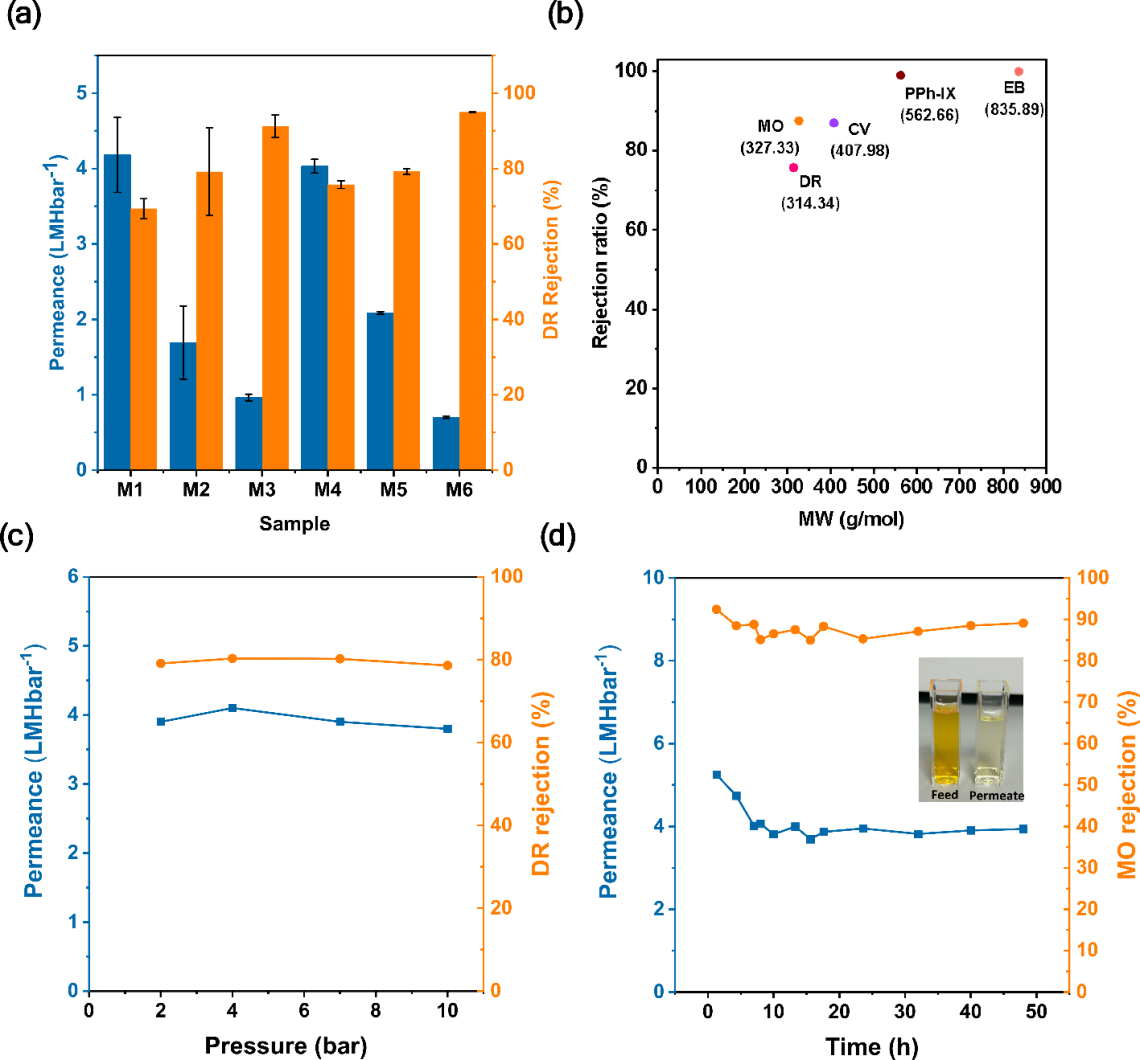
(a) Water permeance and DR rejection of Hyb-PGO/ZIF-8 membranes fabricated under different experimental conditions listed in Table 1. (b) MWCO of the M4 sample tested for different dye molecules. (c) Water permeance and DR rejection of the M4 hybrid membrane under different pressures (dead-end system). (d) Long-term stability test of the M4 membrane at 6 bar and under cross-flow conditions. The inset shows the MO feed solution and collected permeate.

Pristine PGO_5h HF membranes with 5, 15, and 30 s coating times were used to make Hyb-PGO/ZIF-8 membranes (Figure S5 of the Supporting Information). As expected, the PGO HF membrane with a shorter coating time exhibited a lower thickness, resulting in a higher water permeance (Figure S6 of the Supporting Information). Table 1 presents the Hyb-PGO/ZIF-8 membranes prepared under different conditions. Figure 7 displays the water permeance and DR rejection of the Hyb-PGO/ZIF-8 membranes. All hybrid membranes demonstrated better rejection performances than the pristine PGO_5h HF membranes because ZIF-8 crystals selectively patched the defects (Figure 8a and Figure S6 of the Supporting Information). For instance, the DR rejection of the M4 hybrid membrane was 80%, whereas pristine PGO HF showed almost no rejection of the DR (6.5%). This underlines the crucial impact of grown ZIF-8 nanocrystals in boosting the size-sieving performance of the Hyb-PGO/ZIF-8 membranes. Comparing the performance of the M1 and M4 hybrid membranes indicated that a longer immersion time in the metal solution (3 h) resulted in a better rejection performance for the M4 membrane, presumably due to the higher loading of ZIF-8 crystals grown in the laminar structure of the membrane. The surface properties of the hybrid membranes can influence their rejection capabilities. The measured water contact angles of pristine PGO and Hyb-PGO/ZIF-8 membranes were relatively constant, suggesting that the low loading of ZIF-8 nanocrystals does not significantly impact the surface properties (Figure S7 of the Supporting Information). The MWCO of M4 was measured using different dye molecules, including EB, PPh-IX, CV, MO, and DR (Figure 8b). The MWCO was approximately 320, indicating good size-sieving performance of the M4 hybrid membrane. This suggests that crystals grown on the edges of PGO flakes considerably enhanced the size-sieving capability of the hybrid membrane. Furthermore, the M4 hybrid membrane was stable under high pressure (up to 10 bar) without compaction or loss of performance, as shown in Figure 8c. The MO rejection performance of the M4 membrane under cross-flow conditions was evaluated for 48 h, as shown in Figure 8d. The stable water permeance and rejection imply that the bonds created between the ZIF-8 crystals and the PGO nanosheets improved the membrane stability. In addition, the M4 membrane showed no compaction over 24 h, as tested using the dead-end filtration system (Figure S8 of the Supporting Information). The fabrication method and performance of the Hyb-PGO/ZIF-8 HF membranes were compared to those of previous studies (Table S1 of the Supporting Information).^17,21,32,33^ Most GO/ZIF-8 membranes were prepared via simple physical mixing, exhibiting high water permeance but poor stability and rejection performance. This is attributed to ZIF-8 nanocrystals not fully patching the farmwork defects in the GO membranes. Despite the promising water permeance of f-GO@ZIF-8 HF membranes made via ice-templating/*in situ* growth, the framework defects in GO were not completely covered, as indicated by the MWCO of 800 Da for dye molecules. Interestingly, the Hyb-PGO/ZIF-8 HF membrane exhibited 80% rejection for DR and 100% rejection for PPh-IX (a neutral dye), demonstrating better patching of the framework defects.

**Table 1:** Fabrication Conditions for the Hyb-PGO/ZIF-8 Membranes

		metal		ligand	
sample	coating time (s)	solvent	time (h)	solvent	time (h)
M1	5	MeOH	1	MeOH	3
M2	15	MeOH	1	MeOH	3
M3	30	MeOH	1	MeOH	3
M4	5	MeOH	3	MeOH	3
M5	15	MeOH	3	MeOH	3
M6	30	MeOH	3	MeOH	3

**Figure 9:**
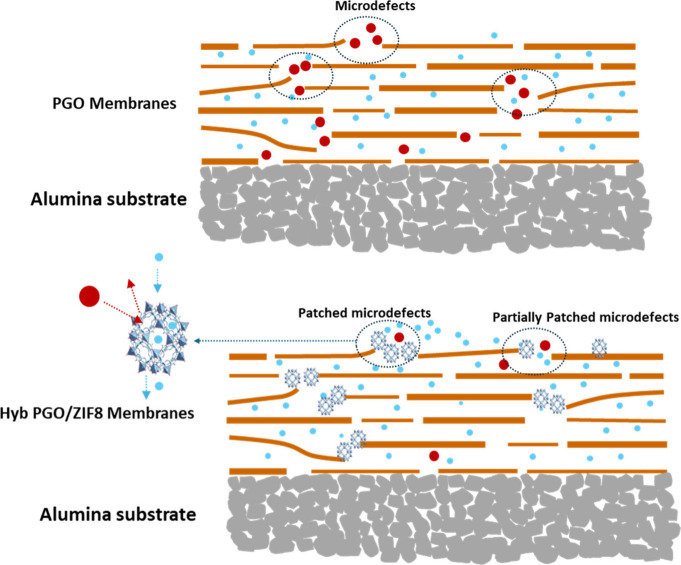
Schematic illustration of the water transport and dye separation mechanism of PGO and Hyb-PGO/ZIF-8 membranes.

Figure 9 schematically illustrates the separation mechanism in the Hyb-PGO/ZIF-8 membrane. The pristine PGO membrane intrinsically includes framework microdefects, which facilitate the non-selective transport of non-desired species through the membrane, leading to relatively poor rejection performance over a prolonged operation. These microdefects provide interconnected pores (pathways) for metal ions and ligand to penetrate through the membrane in coordination and *in situ* growth steps, followed by the formation of ZIF-8 nanocrystals. In comparison to water, using methanol as a solvent can minimize the incorporation of the ZIF-8 precursor into interlayer spacing due to the lower swelling of PGO. This leads to lower permeation resistance induced by possibly the remaining non-coordinated metal ion and linkers, which is reflected by high water permeance of the Hyb-PGO/ZIF-8 membrane relative to the pristine PGO membrane. Therefore, by targeted growth of ZIF-8 nanocrystals in the laminar structure of the membrane, most but not all of these non-selective pathways can be patched. In summary, the targeted growth of ZIF-8 nanocrystals with a small pore size can simultaneously improve the structural stability and size-sieving performance of PGO membranes.

## Conclusion

We successfully fabricated a Hyb-PGO/ZIF-8 membrane through a targeted growth approach that demonstrated a good dye rejection performance and great structural stability for dye nanofiltration. This fabrication technique relies on targeted coordination between the metal part of the ZIF-8 crystals and the hydroxyl groups on the PGO nanosheets, as proven by XPS analysis. Other structural analyses, including XRD, FTIR, and TGA, further validated the targeted growth of crystals in the layered structure of the PGO membrane. The size, loading, and growth behavior of the ZIF-8 nanocrystals were controlled utilizing methanol, the proper solvent, and optimizing the fabrication parameters. The Hyb-PGO/ZIF-8 hybrid membrane (M4) fabricated under optimized conditions (i.e., PGO membrane with the highest permeance, methanol as the solvent, and controlled loading of ZIF-8 nanocrystals) demonstrated stable performance under pressure and prolonged cross-flow operation, indicating robust bonding between the ZIF-8 crystals and the PGO flakes. The MWCO of the resultant membrane improved from 570 to 320 g/mol without losing the water permeance, implying that the framework defects were efficiently patched. Therefore, it can be concluded that the preferential and targeted growth of ZIF-8 nanocrystals minimized their hindrance effect, substantially improving the stability and size exclusion of membranes without compromising the permeance. This research introduces a new approach for precisely patching framework defects to boost the size-sieving ability and stability of GO-based membranes by regulating the interlayer *d* spacing using nanoporous materials. This strategy can be applied to other two-dimensional (2D) materials to improve their structural integrity in the 3D laminar structure.

## Supporting Information Available

SEM images of Hyb-PGO/ZIF-8 hybrid membranes (Figures S1 and S2), N 1s XPS spectra of PGO and Hyb-PGO/ZIF-8 hybrid membranes (Figure S3), XPS depth profile of the Hyb-PGO/ZIF-8 hybrid membrane (Figure S4), surface and cross-sectional SEM images of PGO membranes prepared by different coating times (Figure S5), water permeance and dye rejection of pristine PGO membranes (Figure S6), contact angle of PGO_5h and Hyb-PGO/ZIF-8 membranes (Figure S7), stability of the Hyb-PGO/ZIF-8 membrane tested using the dead-end system (Figure S8), and comparison the water permeance and MWCO of Hyb-PGO/ZIF-8 membranes to GO/ZIF-8 membranes previously reported (Table S1) (PDF)
