# Supplementary material for: Structurally Stable Hollow-Fiber-Based Porous Graphene Oxide Membranes with Improved Rejection Performance by Selective Patching of Framework Defects with Metal–Organic Framework Crystals
Source: ACS Appl Mater Interfaces. 2024 Dec 19;17(1):1803–12. doi: 10.1021/acsami.4c13400 (PMC11783547; doi:10.1021/acsami.4c13400)
Supplement: Supplementary file 1 — am4c13400_si_001.pdf [file am4c13400_si_001.pdf]

Supporting Information

Structurally Stable Hollow Fiber-based Porous Graphene Oxide Membranes with Improved Rejection Performance by Selective Patching of Framework Defects with MOF crystals

*Farhad Moghadam, Chenxi Zhang, Zihao Li, Jianing Li, Mengjiao Zhai, Kang Li**

Barrer Centre, Department of Chemical Engineering, Imperial College London, London, SW7 2AZ, UK

Corresponding authors: kang.li@imperial.ac.uk

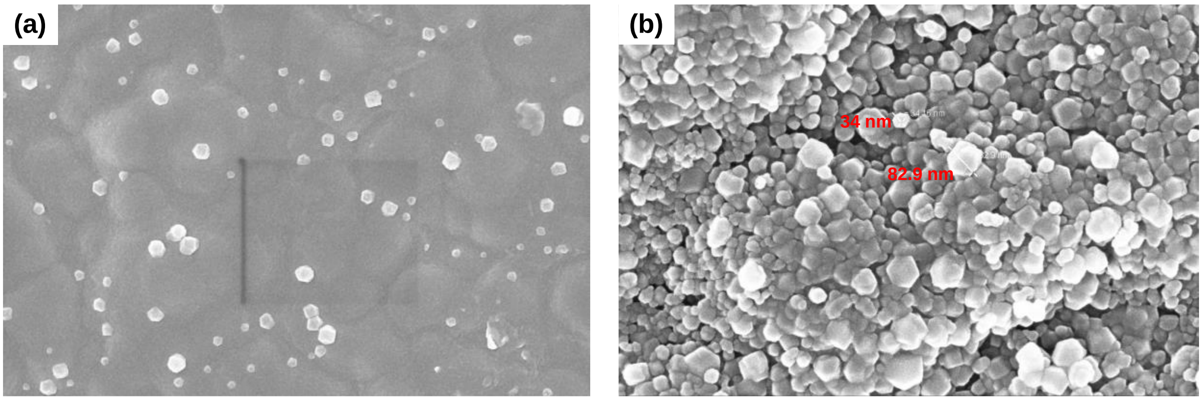

Figure S1. Surface SEM image of (a) Hyb-PGO/ZIF8(M_M) and (b) Hyb-PGO/ZIF8(W_M) membranes. The size of ZIF-8 nanocrystals is in the range of 30-100 nm.

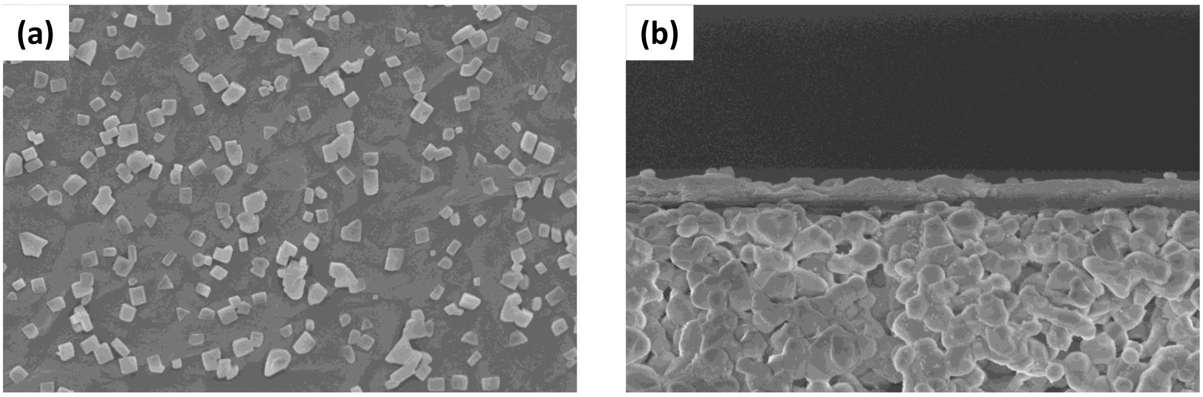

Figure S2. Surface (a) and cross-sectional (b) SEM images of Hyb-PGO/ZIF8 membrane prepared using water as solvent in both steps of ZIF-8 growth. The PGO coating time was 30 and the metal and ligand concentrations in solvents were 3000 ppm and 32000 ppm, respectively.

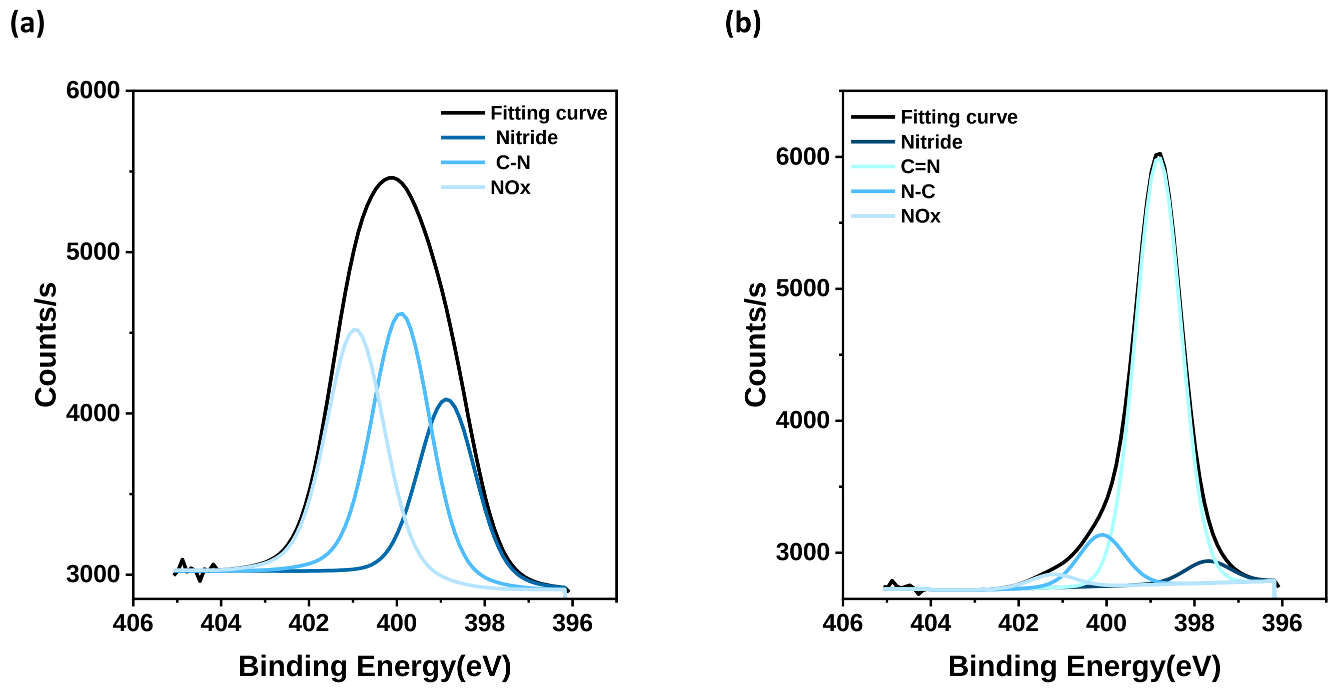

Figure. S3. N 1s spectra of (a) pristine PGO (a) and (b) Hyb-PGO/ZIF8 membranes. The metal and ligand concentrations were 3000 ppm and 32000 ppm, respectively.

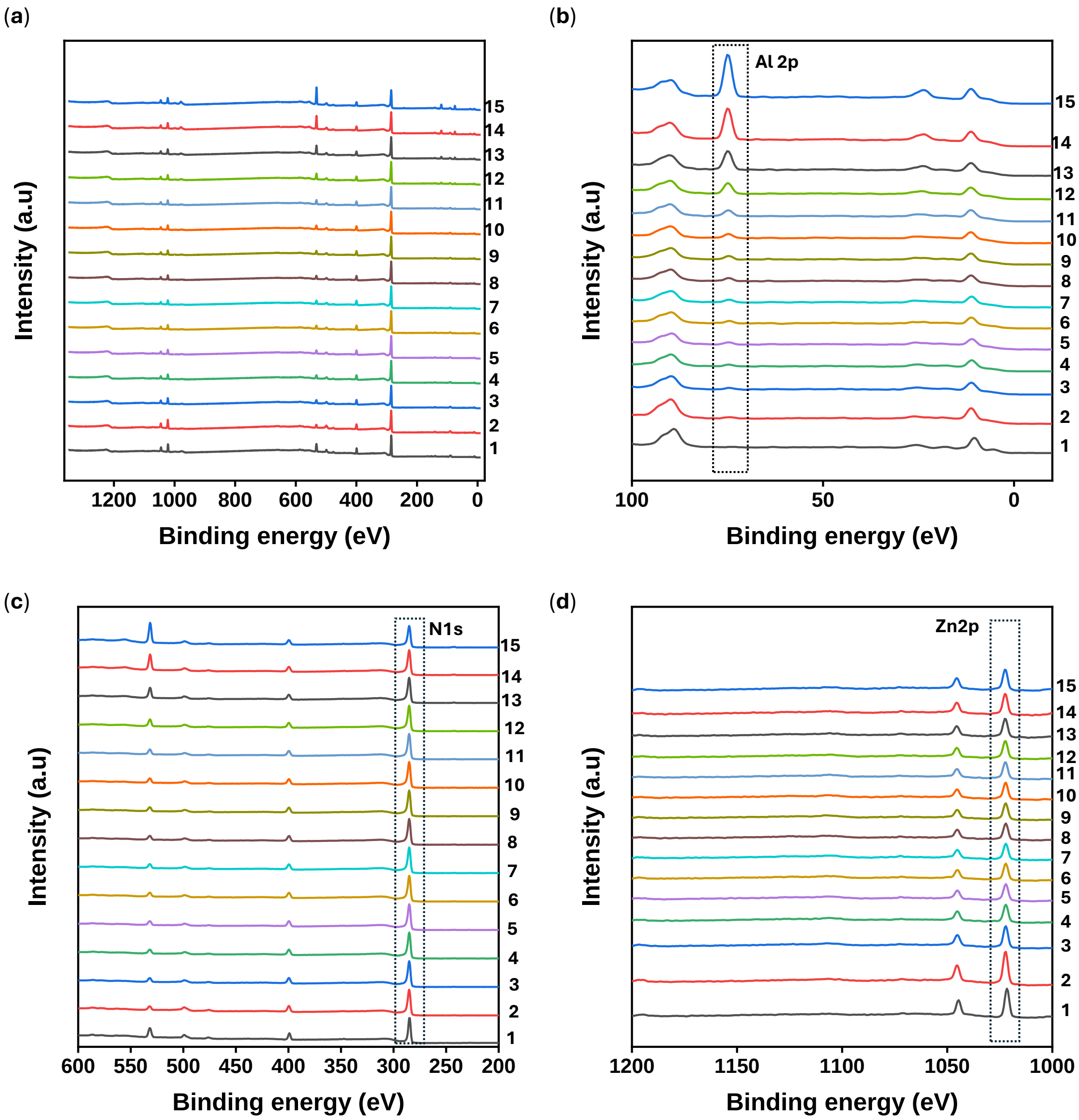

Figure. S4. Depth profile X-ray photoelectron spectroscopy of M4 membrane (a). Magnified (b) Al2p, (c) N1s, and (d) Zn2p spectra across the membrane. Point 1 refers to the surface, points 2-14 are intervals and point 15 is the bottom point close to alumina substrate. The binding energy of Al2p, C1s, N1s, O1s, and Zn2p are 74.5 eV, 285.0 eV, 399.4 eV, 531.7 eV and 1022.1 eV, respectively.

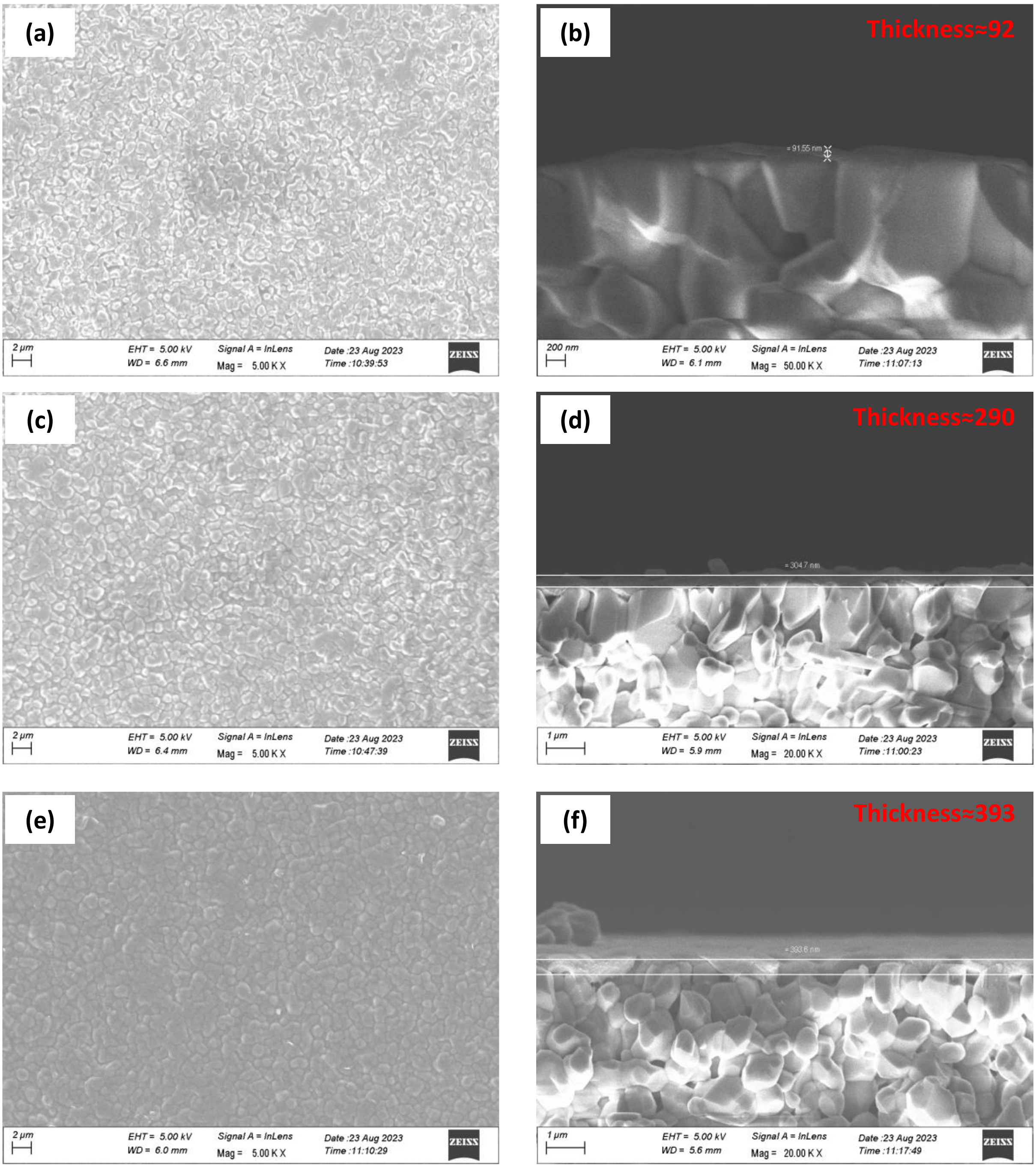

Figure. S5. Surface (a,c,e) and cross-sectional (b,d,f) SEM images of PGO membranes prepared by different coating times. (a),(b) 5 s (c),(d) 15 s and (e),(f) 30 s vacuum coating. The PGO concentration was 0.1 mg/ml.

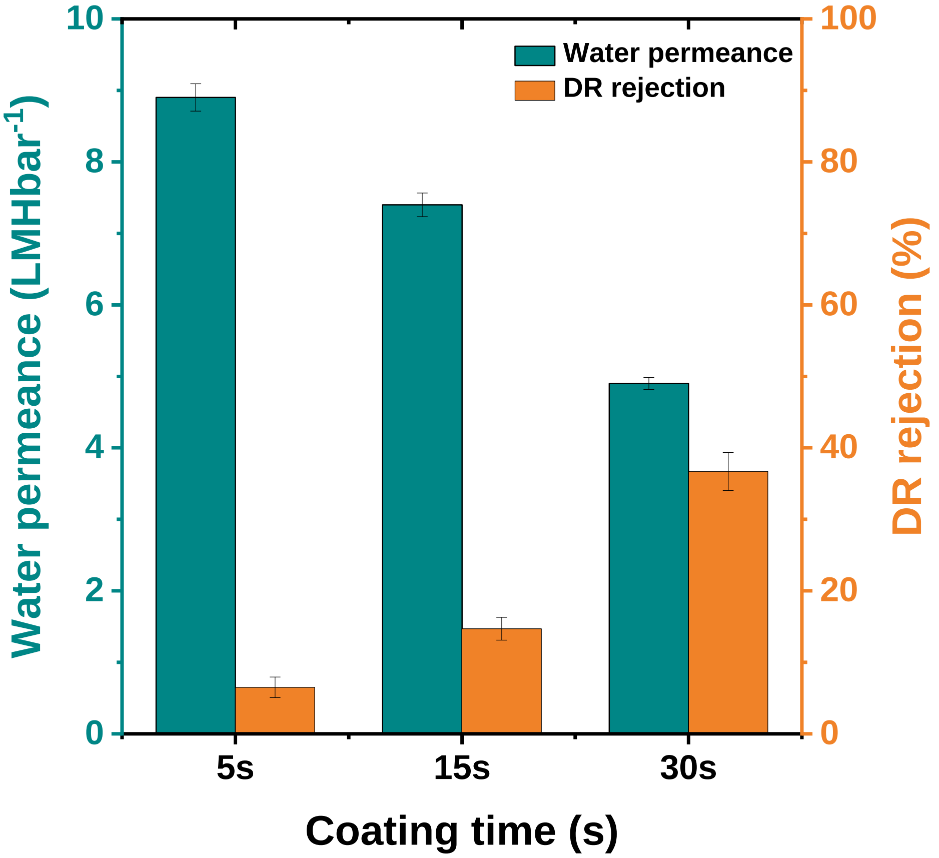

Figure. S6. Water permeance and DR rejection of PGO HF membranes fabricated by different vacuum coating times. The concentration of PGO dispersion was 0.1 mg/ml.

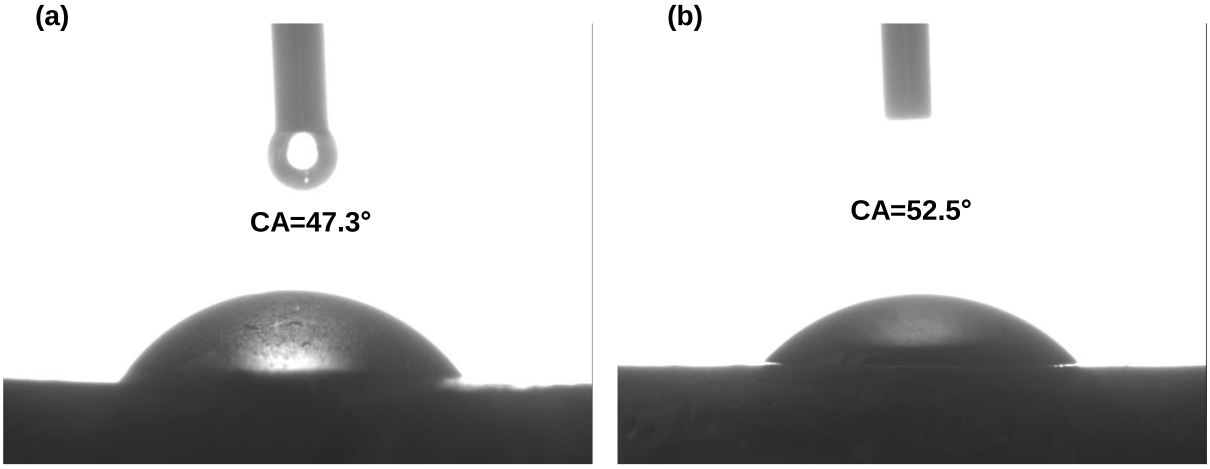

Figure S7. Contact angle of (a) PGO_5h and (b) Hyb-PGO/ZIF8 membranes.

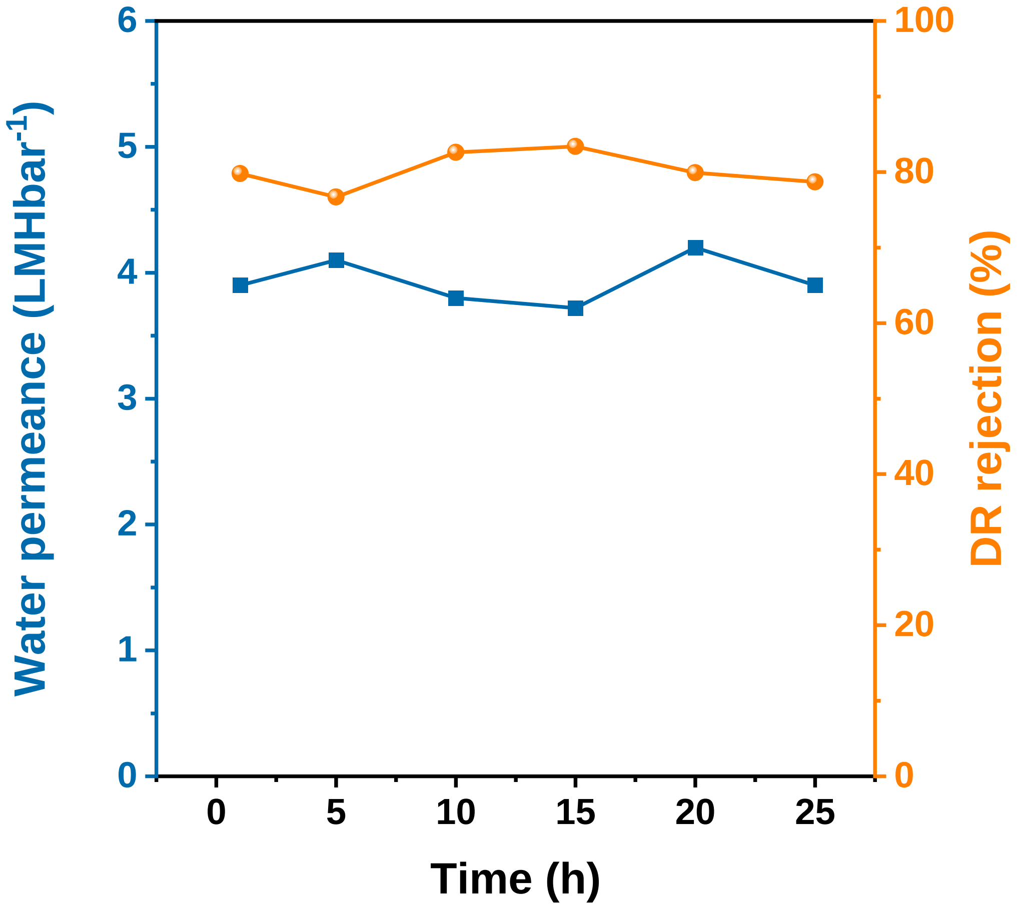

Figure. S8. Stability of M4 hybrid membrane tested using dead-end system for 24 hours.

Table S1: Comparison the water permeance and MWCO of Hyb-PGO/ZIF8 membranes with GO/ZIF-8 membranes previously reported.

Membrane	Fabrication approach	Water permeance $LMHbar^{-1}$	Rejection	Stability	Ref
GO/Cu-TCCP	Physical mixing	100	Poor rejection for neutral dyes (< 20%)	Relatively stable	[1]
GO/etched-ZIF-8	Physical mixing	25-45	MWCO≈700	N/A	[2]
GO/ZIF-8	Physical mixing	≈70	UF range	good	[3]
f-GO@ZIF-8	Ice templating/in situ growth	49.8	MWCO≈800	good	[4]
This work	Selective-coordination/controlled growth	4.1	MWCO≈320 90% for methyl orange (327 Da) and Crystal violet (408 Da) dyes, 80% for neutral DR (314 Da)	good	This work

References:

(1) Zheng Wang, J. Z., Shuainan Xu, Yatao Zhang, Bart Van der Bruggen. Graphene-like MOF nanosheets stabilize graphene oxide membranes enabling selective molecular sieving. *Journal of Membrane Scienc* **2021**, *633* (119397). DOI: https://doi.org/10.1016/j.memsci.2021.119397.
(2) Li, Y.; Zhang, X.; Yang, A.; Jiang, C.; Zhang, G.; Mao, J.; Meng, Q. Polyphenol etched ZIF-8 modified graphene oxide nanofiltration membrane for efficient removal of salts and organic molecules. *Journal of Membrane Science* **2021**, *635*, 119521. DOI: https://doi.org/10.1016/j.memsci.2021.119521.
(3) Makhetha, T. A.; Moutloali, R. M. Stable zeolitic imidazolate framework-8 supported onto graphene oxide hybrid ultrafiltration membranes with improved fouling resistance and water flux. *Chemical Engineering Journal Advances* **2020**, *1*, 100005. DOI: https://doi.org/10.1016/j.ceja.2020.100005.
(4) Zhang, W. H.; Yin, M. J.; Zhao, Q.; Jin, C. G.; Wang, N.; Ji, S.; Ritt, C. L.; Elimelech, M.; An, Q. F. Graphene oxide membranes with stable porous structure for ultrafast water transport. *Nat Nanotechnol* **2021**, *16* (3), 337-343. DOI: 10.1038/s41565-020-00833-9 From NLM PubMed-not-MEDLINE.
